# Characterization of the Sterol 24-C-Methyltransferase Genes Reveals a Network of Alternative Sterol Biosynthetic Pathways in *Mucor lusitanicus*

**DOI:** 10.1128/spectrum.00315-23

**Published:** 2023-04-10

**Authors:** Kitti Bauer, Bence Rafael, Bernadett Vágó, Sándor Kiss-Vetráb, Anna Molnár, Csilla Szebenyi, Mónika Varga, András Szekeres, Csaba Vágvölgyi, Tamás Papp, Gábor Nagy

**Affiliations:** a Department of Microbiology, University of Szeged, Szeged, Hungary; b ELKH-SZTE Fungal Pathomechanisms Research Group, Faculty of Science and Informatics, University of Szeged, Szeged, Hungary; University of Debrecen

**Keywords:** mucormycosis, azole resistance, *erg6* gene, ergosterol, eburicol, zymosterol, CRISPR-Cas9

## Abstract

Certain members of the order *Mucorales* can cause a life-threatening, often-fatal systemic infection called mucormycosis. Mucormycosis has a high mortality rate, which can reach 96 to 100% depending on the underlying condition of the patient. *Mucorales* species are intrinsically resistant to most antifungal agents, such as most of the azoles, which makes mucormycosis treatment challenging. The main target of azoles is the lanosterol 14α-demethylase (Erg11), which is responsible for an essential step in the biosynthesis of ergosterol, the main sterol component of the fungal membrane. Mutations in the *erg11* gene can be associated with azole resistance; however, resistance can also be mediated by loss of function or mutation of other ergosterol biosynthetic enzymes, such as the sterol 24-C-methyltransferase (Erg6). The genome of Mucor lusitanicus encodes three putative *erg6* genes (i.e., *erg6a*, *erg6b*, and *erg6c*). In this study, the role of *erg6* genes in azole resistance of *Mucor* was analyzed by generating and analyzing knockout mutants constructed using the CRISPR-Cas9 technique. Susceptibility testing of the mutants suggested that one of the three genes, *erg6b*, plays a crucial role in the azole resistance of *Mucor*. The sterol composition of *erg6b* knockout mutants was significantly altered compared to that of the original strain, and it revealed the presence of at least four alternative sterol biosynthesis pathways leading to formation of ergosterol and other alternative, nontoxic sterol products. Dynamic operation of these pathways and the switching of biosynthesis from one to the other in response to azole treatment could significantly contribute to avoiding the effects of azoles by these fungi.

**IMPORTANCE** The fungal membrane contains ergosterol instead of cholesterol, which offers a specific point of attack for the defense against pathogenic fungi. Indeed, most antifungal agents target ergosterol or its biosynthesis. Mucormycoses-causing fungi are resistant to most antifungal agents, including most of the azoles. For this reason, the drugs of choice to treat such infections are limited. The exploration of ergosterol biosynthesis is therefore of fundamental importance to understand the azole resistance of mucormycosis-causing fungi and to develop possible new control strategies. Characterization of sterol 24-C-methyltransferase demonstrated its role in the azole resistance and virulence of *M. lusitanicus.* Moreover, our experiments suggest that there are at least four alternative pathways for the biosynthesis of sterols in *Mucor*. Switching between pathways may contribute to the maintenance of azole resistance.

## INTRODUCTION

Members of the *Mucorales* are saprotrophic filamentous fungi, but several species are also known as opportunistic human pathogens (e.g., Lichtheimia corymbifera, Rhizopus delemar, Mucor circinelloides, and Mucor lusitanicus). They can cause an uncommon, life-threatening infection in immunocompromised patients called mucormycosis. The mortality of mucormycosis can reach 96 to 100% depending on underlying disease. Treatment of mucormycosis is extremely challenging due to the different manifestations of the infection (such as cutaneous, pulmonary, or rhino-orbito-cerebral mucormycosis) and the diverse causative agents. Furthermore, treatment often differs in duration and may require multidisciplinary approaches, such as surgery and other symptomatic therapies (1). The main approach is to combine antifungal therapy with surgical interventions, which can increase the survival rate from 30 to 70% (2). For the treatment of mucormycosis, liposomal or lipid formulations of amphotericin B (AmB) are suggested as the first-line antifungal therapy (3). AmB can form a complex with ergosterol and damage the fungal cell membrane by altering its permeability (4, 5). However, the number of antifungal agents for the treatment of mucormycosis is limited, because *Mucorales* species are resistant to the frequently used antifungal agents, such as azoles (e.g., fluconazole or voriconazole) or echinocandins, which are effective in both candidiasis and aspergillosis (6, 7). Posaconazole and isavuconazole are two triazoles which can be useful against *Mucorales* species *in vitro* and *in vivo* (8, 9), and they are approved for second-line or salvage therapy of mucormycosis (3, 9). However, it should also be mentioned that posaconazole and isavuconazole have little or no inhibitory effects on fungi belonging to the *Mucor* genus (10 to 12).

Ergosterol is the main sterol component of the cell membrane in fungal cells, while in mammalian cells the same function is performed by cholesterol. This key difference makes the specific ergosterol biosynthesis a successful target for antifungal drugs (13). The main target of most antifungal agents, such as morpholines, allylamines, and azoles, are the enzymes of the ergosterol biosynthesis pathway (14, 15). The specific target of azoles is a cytochrome P450 enzyme, the lanosterol 14α-demethylase enzyme (Erg11), which mediates a crucial step of the synthesis of ergosterol, the conversion of lanosterol to 4,4-dimethyl-cholesta-8,14,24-trienol. Inhibition of Erg11 may cause the accumulation of toxic 14α-methyl sterols, which alter the stability and permeability of the cell membrane (16). However, resistance to azoles in certain pathogenic fungi, such as in *Candida* species, can also be mediated by loss or mutation of the ergosterol biosynthetic enzymes Erg3 (i.e., sterol C-5 desaturase) and Erg6 (i.e., sterol 24-C-methyltransferase), as well (16–19).

**FIG 1:**
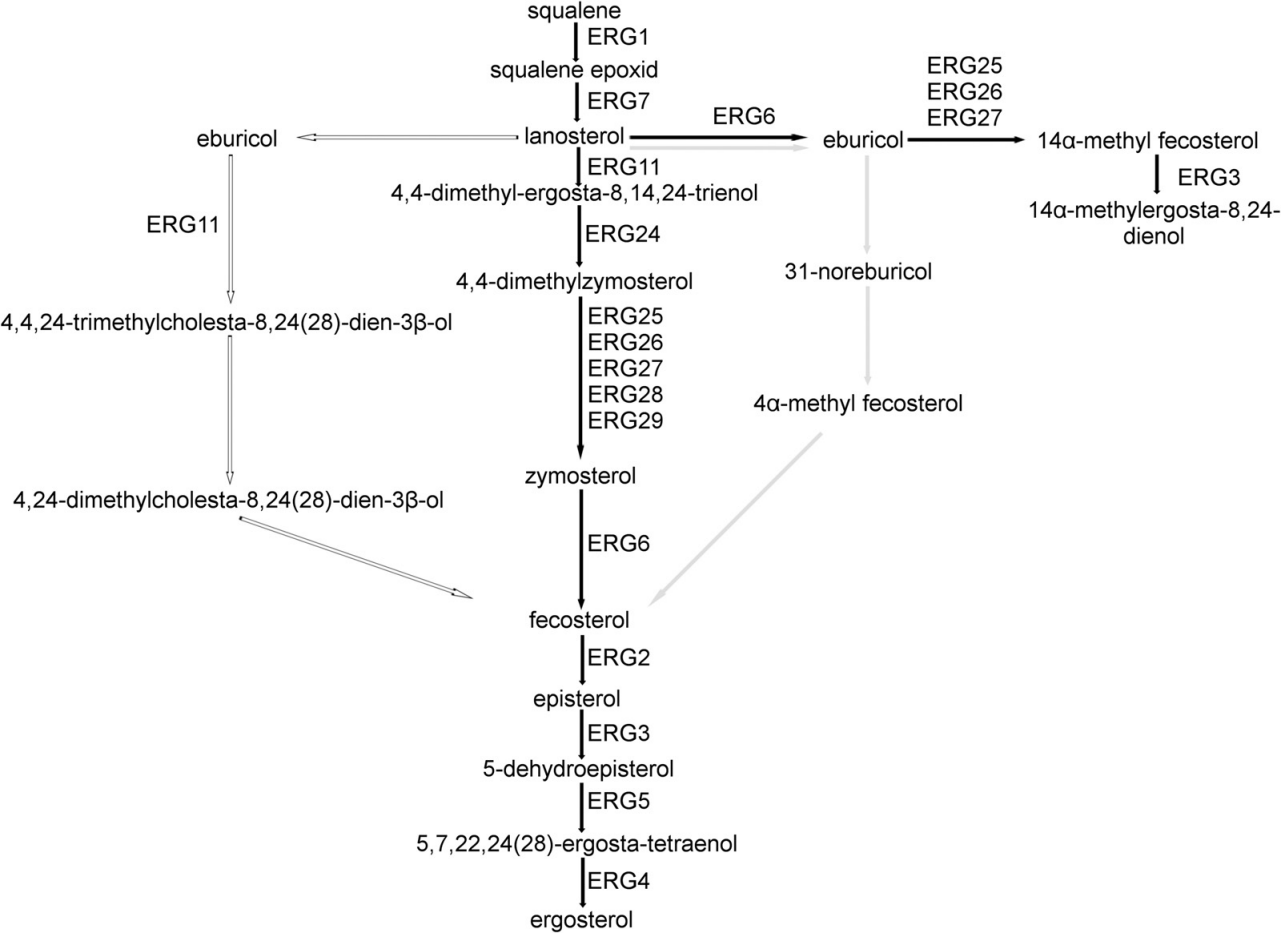
Alternative pathways from lanosterol to fecosterol. The described pathway for Saccharomyces cerevisiae (13) is indicated with black arrows, that for Aspergillus fumigatus (20) is indicated with white arrows, and that for Cryptococcus neoformans (37) is indicated with gray arrows.

Erg6 is the sterol 24-C-methyltransferase which catalyzes a conversion at C-24 of the intermediate compound, zymosterol, to fecosterol in the main ergosterol biosynthetic pathway, and it has a role in the conversion of lanosterol to eburicol in the alternative pathway in Saccharomyces cerevisiae (13). However, in Aspergillus fumigatus, *erg6* catalyzes the conversion of lanosterol to eburicol (20) and, in the cases of Aspergillus spp. and Cryptococcus neoformans, the formation of fecosterol from eburicol (Fig. 1).

In *Mucorales* fungi, the role of Erg6 in azole resistance or a possible alternative biosynthetic pathway is still not known. The goal of this study was to characterize the *erg6* gene and clarify its role in the sterol biosynthesis and azole resistance of *Mucor lusitanicus*. M. lusitanicus is a commonly used model organism for studying various molecular microbiological issues in *Mucorales* (21–31), including the pathomechanisms of mucormycosis-causing fungi (30). In the genome of *M. lusitanicus*, three *erg6* genes (named *erg6a*, *erg6b*, and *erg6c*) can be found, and all of them were involved in the present study.

## RESULTS

### *In silico* analysis of the Erg6 proteins.

In the *Mucor lusitanicus* genome database (DoE Joint Genome Institute; *M. lusitanicus* CBS277.49v2.0; https://mycocosm.jgi.doe.gov/Mucci2/Mucci2.home.html), three Erg6 protein-coding genes were found by a similarity search using the amino acid sequence of S. cerevisiae Erg6 (YML008C), and these genes were named Erg6a, Erg6b, and Erg6c. The nucleotide alignments of *erg6* genes, positions of protospacer sequences, positions of primers used for quantitative PCR (qPCR), and validation of mutants are shown in Fig. S1A and B in the supplemental material. An alignment and a comparison of the three *M. lusitanicus* Erg6 amino acid sequences with Erg6 proteins of clinically relevant *Mucorales* species are shown in Fig. S2. The whole Erg6a protein showed 62.87 and 41.53% amino acid identity to Erg6b and Erg6c, respectively, while Erg6b and Erg6c had 47.65% overall identity to each other (Fig. S2). *M. lusitanicus* Erg6a protein showed 88.26% amino acid identity to Mucor circinelloides Erg6 (protein ID 9624), while interestingly, *M. lusitanicus* Erg6b had more than 80% amino acid identity to Erg6 proteins from several *Mucorales* species (Fig. S2).

### Knockout of *erg6* genes using CRISPR-Cas9.

The genome of *M. lusitanicus* encodes three sterol 24-C-methyltransferases, named Erg6a, Erg6b, and Erg6c. Single disruption of the *erg6a*, *erg6b*, and *erg6c* genes by homology-directed repair resulted in the strains MS12-Δ*erg6a*, MS12-Δ*erg6b*, and MS12-Δ*erg6c*, respectively (Table 1). By the simultaneous disruption of *erg6a* and *erg6c*, the strain MS12-Δ*erg6a*-Δ*erg6c* was created (Table 1). Simultaneous disruption of *erg6b* and *erg6a* or *erg6b* and *erg6c* was carried out several times without success. For each knockout strain, two independently derived mutants were used in the further analyses (Table 1). In the experiments performed to disrupt simultaneously the *erg6b* and *erg6a* or *erg6b* and *erg6c* genes, transformed protoplasts died before colony development. It seems the simultaneous disruption of these genes caused a lethal phenotype.

**TABLE 1:** Features of strains used in this study

Strain	Protein ID(s)	Gene(s)	Genotype	Description
MS12			*leu*^−^ *ura*^−^	Parental strain, leucin and uracil auxotrophic
MS12+*pyrG*	1455390	*pyrG*	*leu*^−^ *ura^+^*	Uracil auxotrophy was complemented, leucin auxotrophic
MS12-Δ*erg6a*/1	74496	*erg6a*	*leu*^−^ *ura^+^*	Deletion of *erg6a,* leucin auxotrophic
MS12-Δ*erg6a*/2	74496	*erg6a*	*leu*^−^ *ura^+^*	Deletion of *erg6a,* leucin auxotrophic
MS12-Δ*erg6b*/1	155859	*erg6b*	*leu*^−^ *ura^+^*	Deletion of *erg6b*, leucin auxotrophic
MS12-Δ*erg6b*/2	155859	*erg6b*	*leu*^−^ *ura^+^*	Deletion of *erg6b*, leucin auxotrophic
MS12-Δ*erg6c*/1	151310	*erg6c*	*leu*^+^ *ura*^−^	Deletion of *erg6c,* uracil auxotrophic
MS12-Δ*erg6c*/2	151310	*erg6c*	*leu*^+^ *ura*^−^	Deletion of *erg6c,* uracil auxotrophic
MS12-Δ*erg6a*-Δ*erg6c*/1	74496, 151310	*erg6a* and *erg6c*	*leu*^+^ *ura^+^*	Deletion of *erg6a* and *erg6c*, prototrophic
MS12-Δ*erg6a*-Δ*erg6c*/2	74496, 151310	*erg6a* and *erg6c*	*leu*^+^ *ura^+^*	Deletion of *erg6a* and *erg6c*, prototrophic

### Effect of *erg6* knockout on relative transcript levels of other *erg* genes.

**FIG 2:**
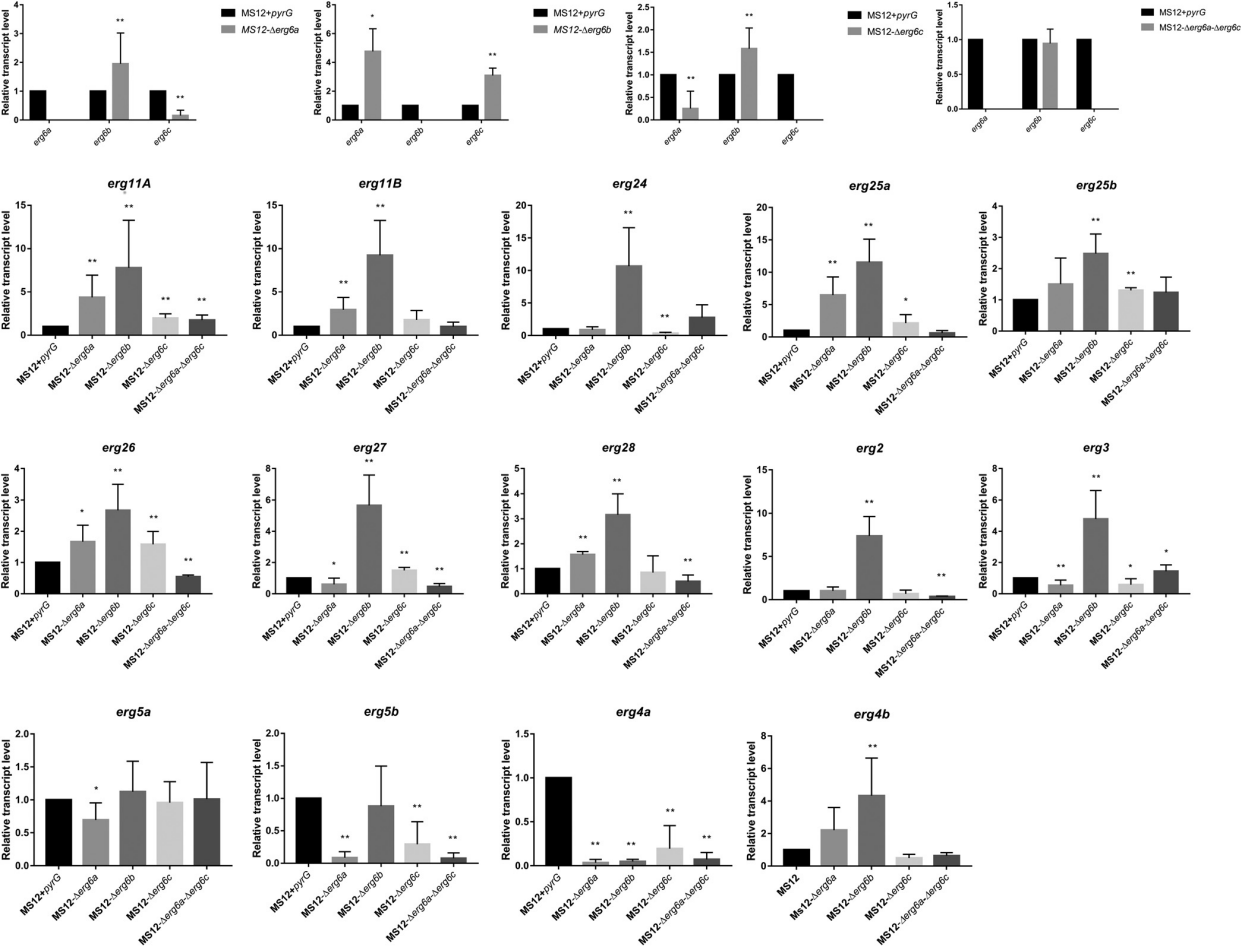
Relative transcript levels of the ergosterol biosynthesis genes in the *erg6* knockout mutant strains. Strains were grown on YNB medium at 25°C; the transcript level of each gene measured in the MS12+*pyrG* control strain was taken as 1. The presented values are averages of three independent experiments of two independent isolates per mutant; error bars indicate standard deviations. Relative transcript values followed by asterisks were significantly differed from the untreated control according to the unpaired *t* test (*, *P* < 0.05; **, *P* < 0.01).

Reverse transcription-quantitative PCR (qRT-PCR) analysis proved the absence of the transcript of the deleted *erg6* genes and revealed that the relative transcript levels of *erg6a* and *erg6c* were significantly increased in MS12-Δ*erg6b*, while single deletion of *erg6a* or *erg6c* resulted in a significantly increased transcript level of *erg6b* (Fig. 2). Interestingly, simultaneous deletion of *erg6a* and *erg6c* had no effect on the relative transcript level of *erg6b* in MS12-Δ*erg6a*-Δ*erg6c*.

Relative transcript levels of *erg11A*, *erg11B*, *erg24*, *erg25a*, *erg25b*, *erg26*, *erg27*, *erg28*, *erg2*, *erg3*, and *erg4b* significantly increased after deletion of the *erg6b* gene (Fig. 2). The lack of *erg6* genes (single or simultaneous deletions of them) resulted in significantly decreased transcript levels of *erg4a*. Relative transcript levels of *erg5b* significantly decreased after single or simultaneous knockout of *erg6a* and *erg6c*, while a decrease in the transcript level of *erg5a* was caused only by the lack of *erg6a*. The lack of *erg6a* or *erg6c* resulted significantly increased transcript levels of *erg11A*, *erg25a*, and *erg26* (Fig. 2).

### Effect of *erg6* knockout on colony growth.

**FIG 3:**
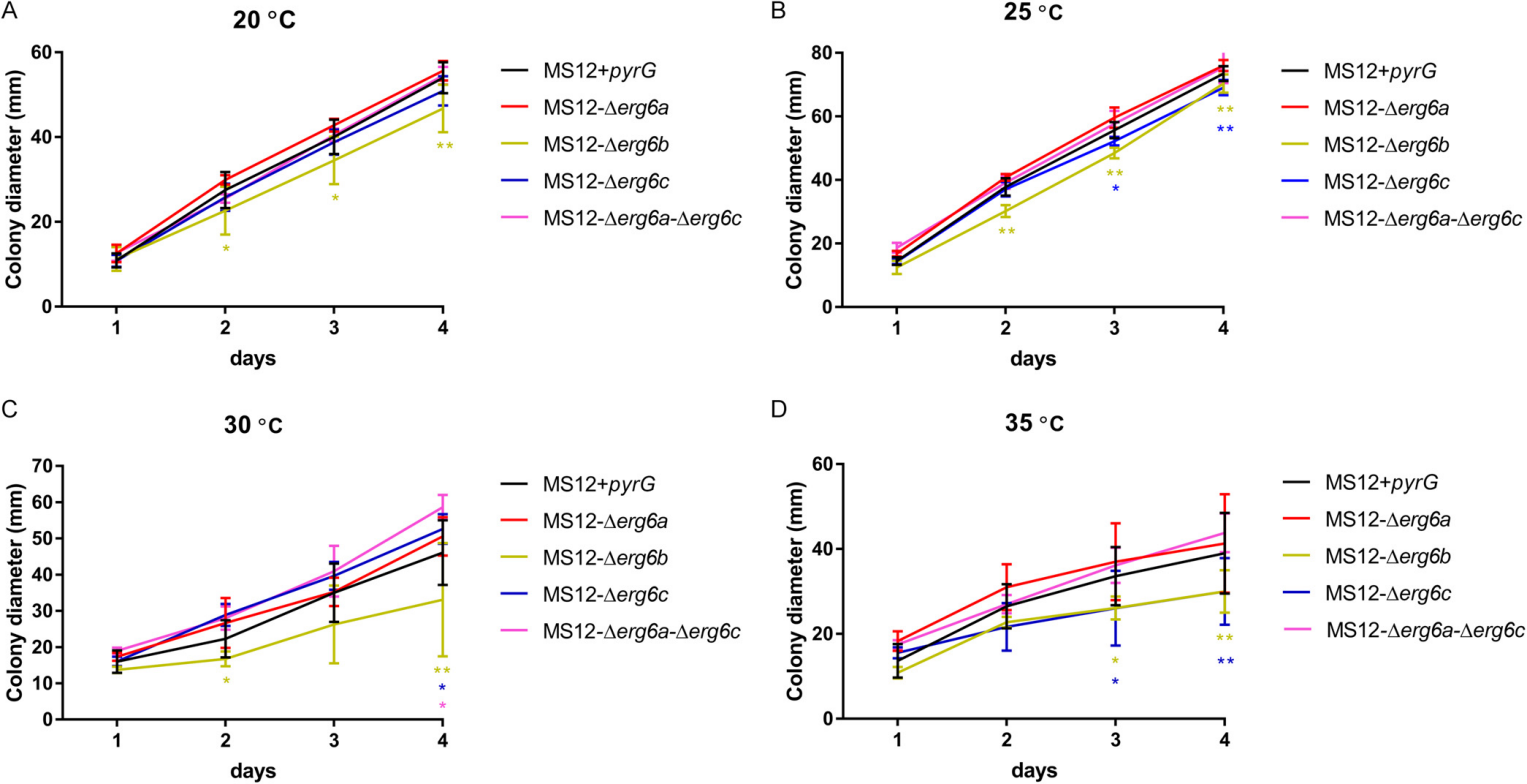
Colony diameters of the *erg6* deletion mutants and the MS12+*pyrG* strain of *Mucor lusitanicus* at 20°C (A), 25°C (B), 30°C (C), and 35°C (D) on YNB medium. The presented values are averages; colony diameters were measured during three independent cultivations of two isolates per mutant (error bars indicate standard deviations). Values followed by asterisks were significantly different from the corresponding value of the MS12 strain according to two-way ANOVA; (*, *P* < 0.05; **, *P* < 0.01).

Growth ability of the *erg6* mutants was examined at 20, 25, 30, and 35°C for 4 days (Fig. 3). Growth analysis was performed with two independent isolates for each *erg6* knockout mutant, with technical and biological triplicates. Deletion of *erg6a* had no effect on the growth ability of MS12-Δ*erg6a* at the examined temperatures. However, the colony diameter of MS12-Δ*erg6b* was significantly decreased at 20, 25, 30, and 35°C (Fig. 3). Interestingly, the colony diameter of MS12-Δ*erg6c* was also significantly decreased at 25 and 35°C (Fig. 3).

**FIG 4:**
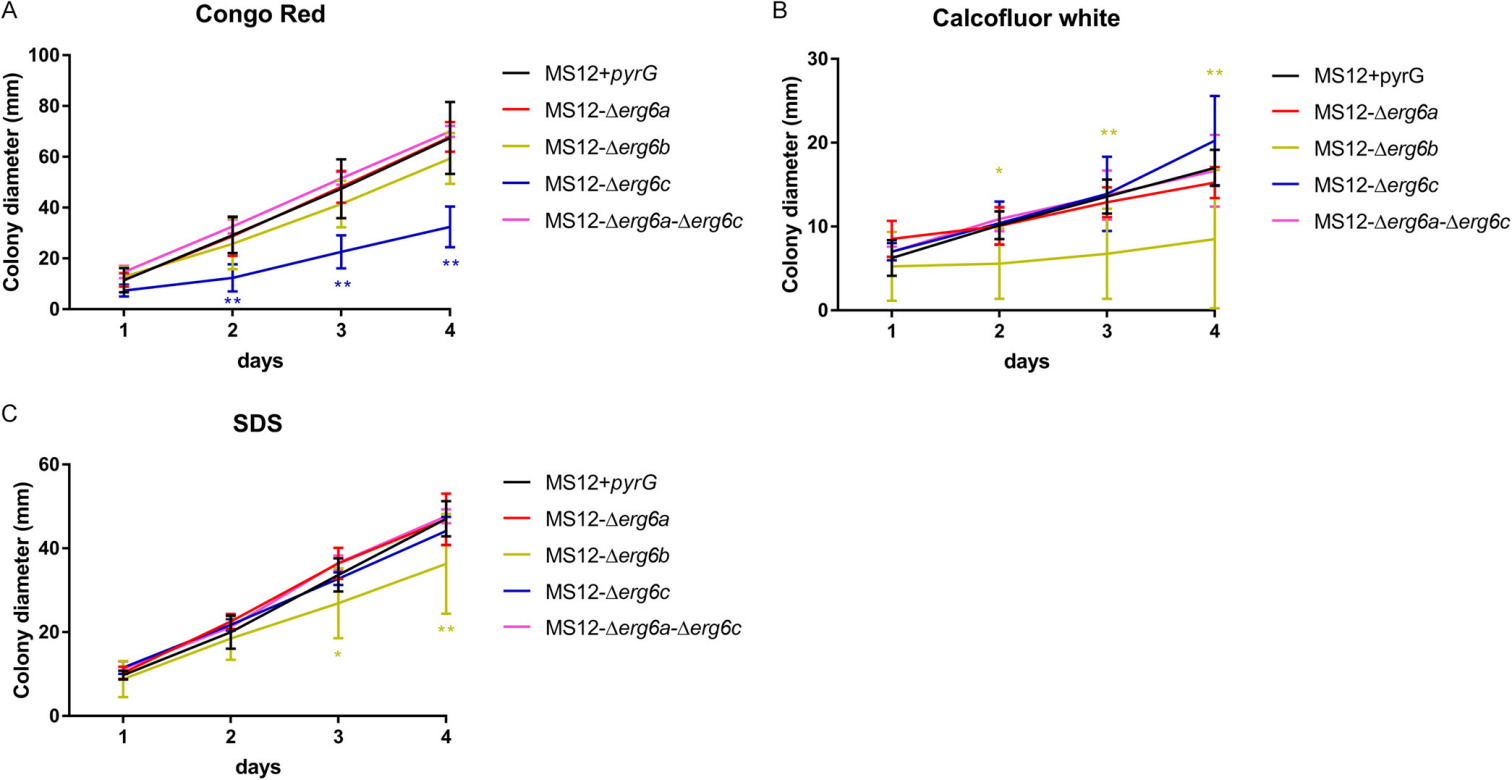
Effects of different stressors on growth abilities of *erg6* mutants and MS12+*pyrG* strain. (A) Effect of Congo red (CR) on the growth of strains. (B) Effect of calcofluor white (CFW) on the growth of strains. (C) Effect of SDS on the growth of strains. The presented values are averages; colony diameters were measured during three independent cultivations of two independent isolates per mutant (error bars indicate standard deviations). Values followed by asterisks significantly differed from the corresponding value of the MS12 strain according to the two-way ANOVA (*, *P* < 0.05; **, *P* < 0.01).

Colony growth of the *erg6* mutants was also investigated in the presence of the cell wall stressors Congo red (CR) and calcofluor white (CFW), as well as the cell membrane stressor SDS (Fig. 4). The colony diameter of MS12-Δ*erg6b* significantly decreased after CFW and SDS treatment (Fig. 4B and C), while the lack of *erg6c* resulted in a significantly decreased colony diameter in the presence of CR (Fig. 4A). The tested stressors had no effect on the growth ability of MS12-Δ*erg6a* (Fig. 4).

### Effect of *erg6* genes on sporulation of *M. lusitanicus*.

**FIG 5:**
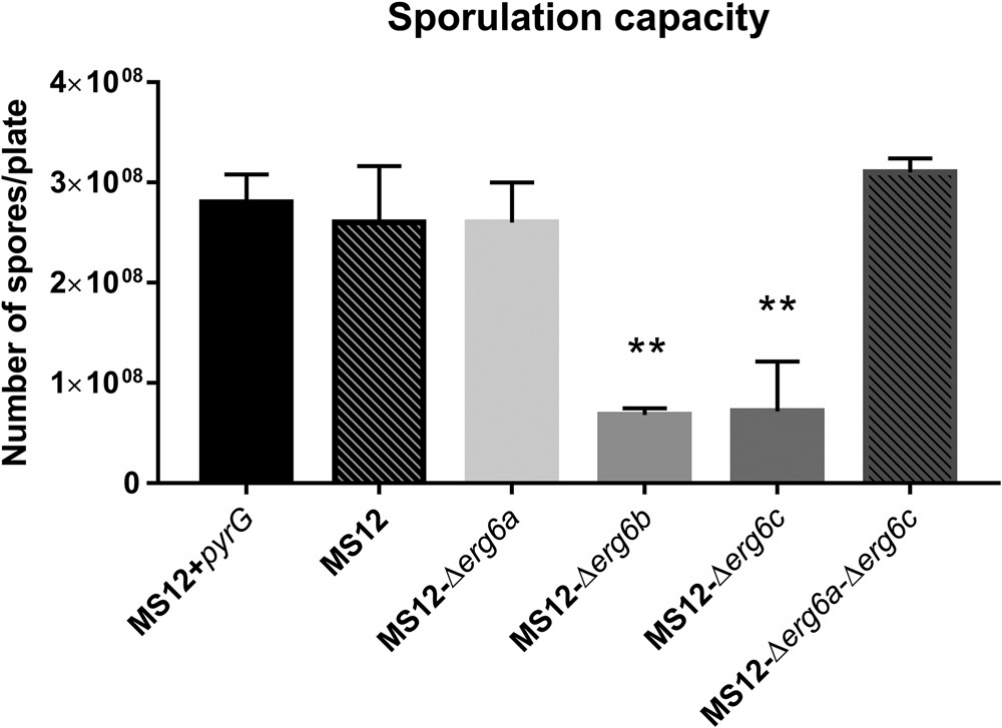
Spore production after 7 days on malt extract agar. The presented values are averages; spores were counted from three independent cultivations of two independent isolates per mutant (error bars indicate standard deviations). Values followed by asterisks significantly differed from the corresponding value of the MS12+*pyrG* strain according to the unpaired *t* test (**, *P* < 0.01).

MS12-Δ*erg6b* and MS12-Δ*erg6c* produced significantly fewer spores than the control strain (MS12+*pyrG*), but this phenotype was not observed in the case of the other *erg6* mutants (Fig. 5). Under a stereomicroscope, sporangia of MS12-Δ*erg6b* had a yellowish color, in contrast to the other tested strains, which produced brownish sporangia (Fig. S3). This yellowish color may indicate that spore formation and maturation are disturbed in this mutant strain.

### Effects of *erg6* genes on susceptibility to azoles and amphotericin B.

Sensitivities of the *erg6* mutants to different azoles and AmB were determined by using a broth microdilution assay, and the MICs of the antifungal agents were determined (Table 2). Knockout of *erg6b* increased the sensitivity of the fungus to ketoconazole, itraconazole, posaconazole, ravuconazole, and isavuconazole, while the susceptibilities of MS12-Δ*erg6a*, MS12-Δ*erg6c*, and MS12-Δ*erg6a-*Δ*erg6c* to all tested azoles were similar to that of the MS12+*pyrG* strain used as a control (Table 1).

**TABLE 2:** MICs of the azoles and AmB against the *erg6* mutants^*a*^

Strain	MIC (μg/mL)						
	Ketoconazole	Itraconazole	Fluconazole	Posaconazole	Ravuconazole	Isavuconazole	AmB
MS12+*pyrG*	>16	>16	>16	4	>16	>16	2
MS12-Δ*erg6a*	>16	>16	>16	4	>16	>16	2
MS12-Δ*erg6b*	0.5	1	>16	0.5	0.5	0.5	2
MS12-Δ*erg6c*	>16	>16	>16	4	>16	>16	2
MS12-Δ*erg6a*-Δ*erg6c*	>16	>16	>16	4	>16	>16	1

a There were two independent isolates per mutant. The control the MS12+*pyrG* strain. The two values reported for the MS12-Δ*erg6b* strain against some of the azoles.

### *In vivo* virulence of *erg6* mutants.

**FIG 6:**
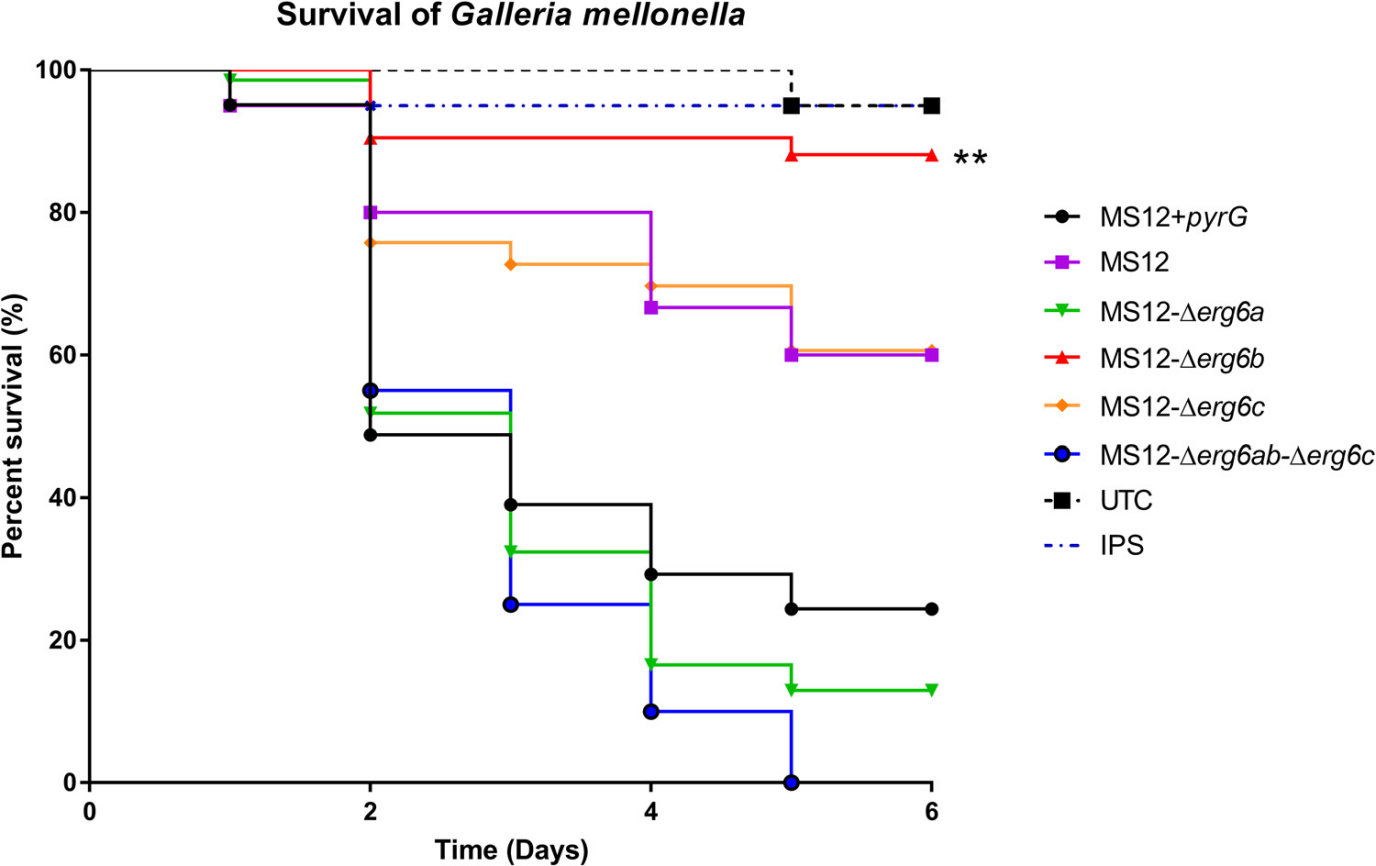
Survival of Galleria mellonella (*n* = 20) infected with the *erg6* mutants or the control *M. lusitanicus* MS12+*pyrG* and MS12 strains. The presented values are averages; survival curves were determined from three independent cultivations of two independent isolates per mutant. Survival curves followed by asterisks significantly differed from the control strain according to the log-rank (Mantel-Cox) test (**, *P* ≤ 0.01). The results summarize the results of 3 independent experiments.

Effect of the *erg6* gene knockout on virulence was examined in a Galleria mellonella *in vivo* invertebrate model (Fig. 6). Knockout of *erg6b* significantly decreased the virulence of the fungus compared to the other the mutants and the control strains. Although the growth ability of MS12-Δ*erg6b*, MS12-Δ*erg6c*, and MS12-Δ*erg6a-*Δ*erg6c* significantly decreased at 30°C compared to that of the MS12+pyrG strain, only the virulence of MS12-Δ*erg6b* decreased significantly. Virulence of the MS12-Δ*erg6c* strain showed the same virulence as the MS12 strain. The MS12-Δ*erg6c* strain is uracil auxotrophic (Table 1), as MS12 strain is, and uracil auxotrophy may affect virulence.

### Sterol composition of *erg6* mutants.

**FIG 7:**
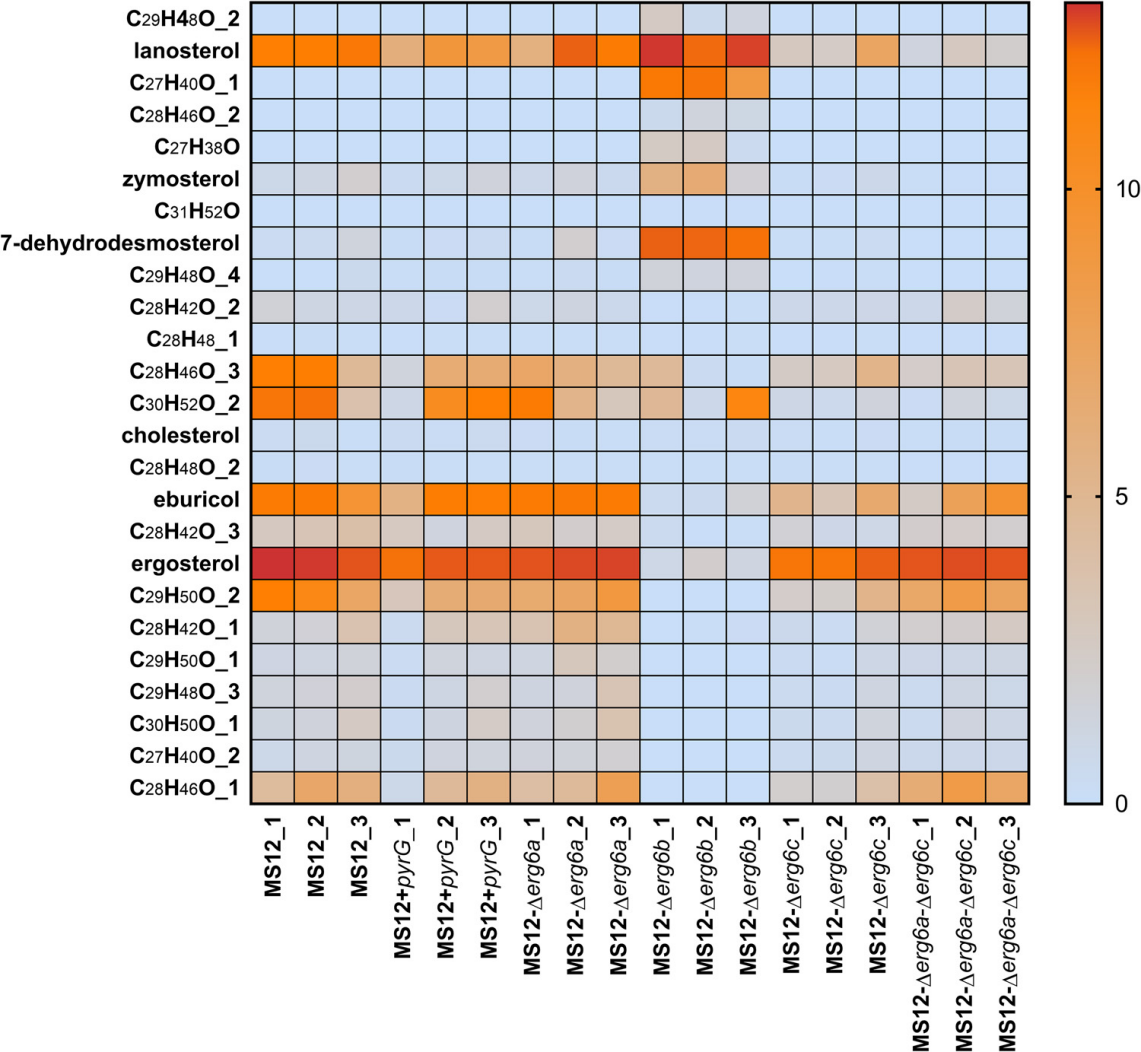
Heat map of sterol composition of different *Mucor* strains. Scale represents the ratio of peak area and internal standard (IS) peak area values. The baseline was set to 1.

**FIG 8:**
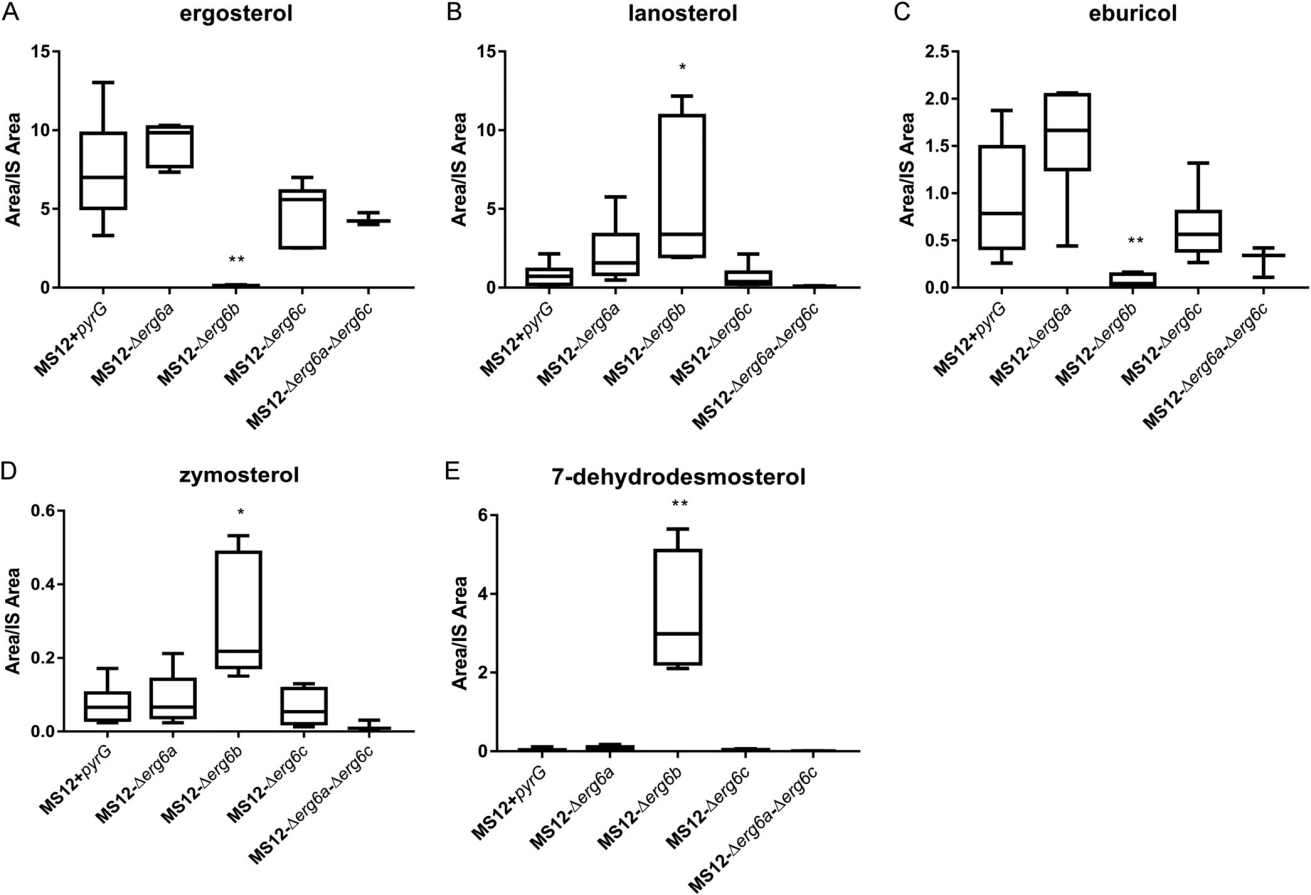
Sterol composition of *erg6* and MS12+*pyrG* strains. The presented values are averages; amounts of sterols were measured from three independent cultivations of two independent isolates per mutant (error bars indicate standard deviations). Values followed by asterisks significantly differed from the corresponding value of the MS12+*pyrG* strain according to the unpaired *t* test (*, *P* < 0.05; **, *P* < 0.01).

Sterol composition of the original MS12 strain and the *erg6* mutants was determined by using a high-performance liquid chromatography–high-resolution mass spectrometry (HPLC-HRMS) analysis. A heat map was generated to summarize the results of the sterol analysis (Fig. 7), which revealed a strikingly different sterol composition in the MS12-Δ*erg6b* mutant compared to the other tested strains. The original MS12 strain produced ergosterol in an amount typical of the wild type (Fig. 8). In addition, larger quantities of eburicol and zymosterol and a small amount of lanosterol were detected. In contrast, in the MS12-Δ*erg6b* strain, the ergosterol content was minimized, as expected (C_28_H_44_O) (Fig. 8A). The amount of the intermediate eburicol also decreased in this strain (Fig. 8C), but the amount of lanosterol and zymosterol increased (Fig. 8B and D). In addition, 7-methyldesmosterol (Fig. 8E) appeared as a new component. In the case of the MS12-Δ*erg6a* and MS12-Δ*erg6c* mutants, similar sterol profiles were detected as in the MS12+*pyrG* strain (Fig. 8). HPLC-HRMS analysis revealed a small amount of cholesterol in the tested strains, but there was no significant difference between the amounts in the tested strains (Fig. 7).

**FIG 9:**
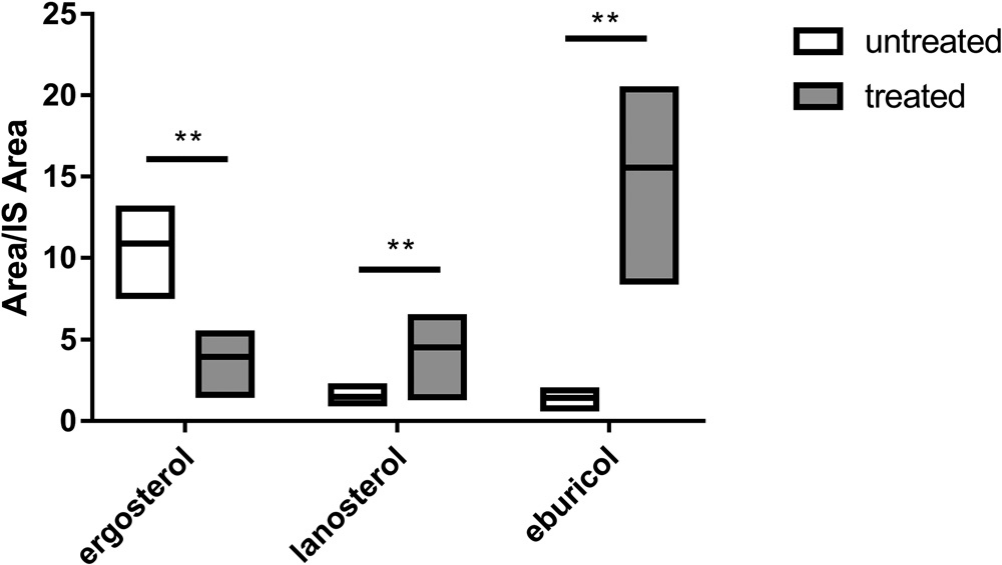
Sterol composition of MS12+*pyrG* and *cyp51* knockout mutant strains. The presented values are averages; amounts of sterols were measured from three independent cultivations of two independent isolates per mutant (error bars indicate standard deviations). Values followed by asterisks significantly differed from the corresponding value of the MS12+*pyrG* strain according to the unpaired *t* test (*, *P* < 0.05; **, *P* < 0.01).

Since Erg11 is the main target of azoles and its catalytic action precedes that of Erg6 in the pathway, the blocking of Erg11 may affect the accumulation of the intermediate compounds and the direction of the biosynthetic pathway. Therefore, the sterol composition was also analyzed by HPLC-HRMS analysis after posaconazole exposure (2 mg/mL in RPMI 1640). Ergosterol content significantly decreased after the posaconazole treatment, while the lanosterol and eburicol contents significantly increased (Fig. 9). Inhibition of Erg11 resulted the accumulation of a compound, the molecular mass and composition of which corresponded to those of 14α-methylfecosterol (C_29_H_48_O).

### Relative transcript levels of DHCR and CDI genes.

**FIG 10:**
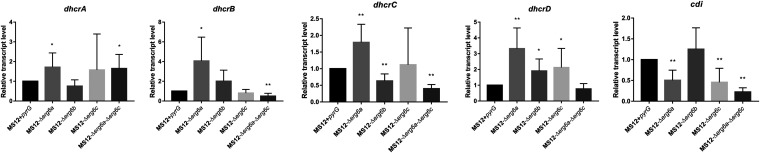
Relative transcript levels of DHCR and CDI genes in the *erg6* knockout mutant strains. Strains were grown on YNB medium at 25°C; the transcript level of each gene measured in the MS12+*pyrG* control strain was taken as 1. The presented values are averages of three independent experiments of two independent isolates per mutant; error bars indicate standard deviations. Relative transcript values followed by asterisks significantly differed from the untreated control according to the unpaired *t* test (*, *P* < 0.05; **, *P* < 0.01).

Analysis of sterol composition of *erg6b* mutants revealed the accumulation of zymosterol and 7-dehidrodesmosterol in the MS12-Δ*erg6b* strain and a small amount of cholesterol in all tested strains. Based on the data available in the KEGG database (https://www.genome.jp/pathway/map00100), homologous sequences of delta24-sterol reductase (DHCR) (1.3.1.72) and cholestenol delta-isomerase (CDI) (5.3.3.5) were searched on the *M. lusitanicus* genome database. DHCR and CDI are responsible for conversion of zymosterol to cholesta-8-en-3β-ol and cholesta-7,24-dien-3β-ol, respectively. Four putative DHCR genes (*dhcrA*, *dhcrB*, *dhcrC*, and *dhcrC*) and one CDI gene (*cdi*) were found. In the MS12-Δ*erg6a* strain, all of the *dhcr* genes proved to be upregulated, while the lack of *erg6c* resulted in increased transcript levels of *dhcrA* and *dhcrD* (Fig. 10). However, due to the lack of *erg6b*, only the relative transcript level of *dhcrD* was significantly increased. Relative transcript levels of *cdi* significantly decreased after knockout of *erg6a* and/or *erg6c* genes, while the lack of *erg6b* had no effect on the expression of the *cdi* gene (Fig. 10).

## DISCUSSION

The genome of *M. lusitanicus* encodes three *erg6* genes, and our results suggested that *erg6b* plays the decisive role in the late ergosterol biosynthetic pathway among these three. The lack of this gene resulted in significantly decreased ergosterol and increased zymosterol content in the cell. Furthermore, knockout of *erg6b* resulted in altered growth ability and increased sensitivity to azoles, and simultaneous deletion of this gene together with *erg6a* or *erg6c* proved to be lethal. Based on molecular phylogenetic studies, it can be suggested that a genome duplication occurred in an ancestor of the *Mucorales* (32, 33), and expansion of the genes is a characteristic feature of these fungi. Thus, several genes are present in multiple copies in their genomes. Usually one of these genes shows significant activity and it is dominant over the other isogenes (33, 34). Apparently, the other two enzymes can compensate to some extent for the lack of Erg6b expression. This compensatory effect was also observed in cases of other paralogous genes of *Mucor*, such as for *hmgr* and *pdr* (34, 35). Knockout of the *erg6* genes affected the expression of other sterol biosynthetic genes, including significantly increased transcription levels of the genes encoding the enzymes, which catalyze the subsequent steps after formation of the intermediate compounds, lanosterol or eburicol (Fig. 8). At the same time, transcription levels of *erg4* and *erg5*, which encode the enzymes catalyzing the last two steps toward the formation of ergosterol, decreased. In Candida albicans, the lack of *erg6* resulted increased transcript levels of certain ergosterol genes, such as *erg7*, *erg11*, *erg25*, and *erg2* (36).

Deletion of the *Mucor erg6b* gene caused a significant decrease in the ergosterol content. Maybe because of the altered sterol composition in the membranes, *erg6b* knockout was associated with reduced growth, especially at higher temperatures, impaired sporulation, and increased sensitivity to cell wall and membrane stressors. In Cryptococcus neoformans, the lack of *erg6* also resulted in an altered growth ability at 30 and 35°C (37), and similar phenotypic changes were observed in the case of Candida lusitaniae (38).

The lack of *erg6* in Kluyveromyces lactis caused reduced ergosterol content in the cells and an impairment in the stress adaptation of the fungus (39). In our study, CW, CR, and SDS were used to induce cell wall and membrane stresses. The lack of *erg6b* expression significantly reduced the colony diameter of the MS12-Δ*erg6b* mutant, suggesting that *erg6b* participates in the maintenance of the normal cell wall and membrane structure in *Mucor.* Growth impairment in the presence of SDS and CR after disruption of *erg6* was also observed in Cryptococcus (37).

In *Ascomycetes*, Erg6 has also been found to play a role in the resistance of different antifungal agents, such as azoles and amphotericin B. A comprehensive study of clinical Aspergillus fumigatus strains showed that certain azole-resistant isolates had mutations in the *hmg1* gene, encoding 3-hydroxy-3-methylglutaryl coenzyme A reductase, and *erg6* (40, 41). Both genes are involved in ergosterol biosynthesis (40, 41). Furthermore, the A. fumigatus *erg6* gene showed strong upregulation after azole exposure. Disruption of *erg6* also caused an increased susceptibility to several azoles, such as itraconazole, fluconazole, and ketoconazole in C. neoformans (37). In *Candida*, deletion of *erg6* also caused high sensitivity to fluconazole (38). In. *M. lusitanicus*, sensitivity of an *erg6* knockout strain, MS12-Δ*erg6b*, to ketoconazole, itraconazole, posaconazole, ravuconazole, and isavuconazole significantly increased. However, the lack of the gene had no effect on the susceptibility of the fungus to fluconazole. In a previous study, deletion of pleiotropic drug resistance transporter genes also caused increased susceptibility to various azoles, except for fluconazole (35). It can be assumed that fluconazole cannot enter into the fungal cells, or immediately after uptake it is exported outside the cell by one or more transporter proteins. Several mechanisms have been described in fluconazole resistance which involve genes of the ergosterol biosynthesis pathway (*erg11* or *erg3*) or involve different drug transporters, such as ABC transporters (e.g., CDR1, CDR2, SNQ2, ABC1) or MFS transporters (e.g., MDR1, TPO3) (42, 43).

MS12-Δ*erg6b* mutant strains showed significantly reduced virulence in the G. mellonella model, suggesting that expression of this gene and/or the sterol composition affects the pathogenicity of *Mucor*. In the case of C. neoformans, deletion of *erg6* also resulted in significantly reduced virulence in *Galleria* and a decreased survival after phagocytosis in an *in vitro* interaction with macrophages (37). MS12-Δ*erg6c* mutant strains showed significantly reduced virulence compared to the MS12+pyrG strain, but the virulence of this strains was similar to that of the MS12 strain. MS12-Δ*erg6c* is a uracil auxotrophic strain, and uracil auxotrophy may have an effect on virulence of *Mucor*, similar to that reported in *Rhizopous microsporus*, where uracil biosynthesis is essential for virulence (44).

The sterol contents of the original *M. lusitanicus* strain as well as those of the *erg6* knockout mutants were analyzed in detail. The fact that lanosterol, zymosterol, and eburicol could be detected in the original strain suggested that ergosterol biosynthesis occurs on two main parallel pathways via either zymosterol or eburicol, as indicated in Fig. 9. The presence of these alternative pathways was strengthened by the increased lanosterol and eburicol contents after posaconazole exposure, as well as the increased lanosterol and zymosterol and decreased eburicol contents of the *erg6b* knockout strains.

**FIG 11:**
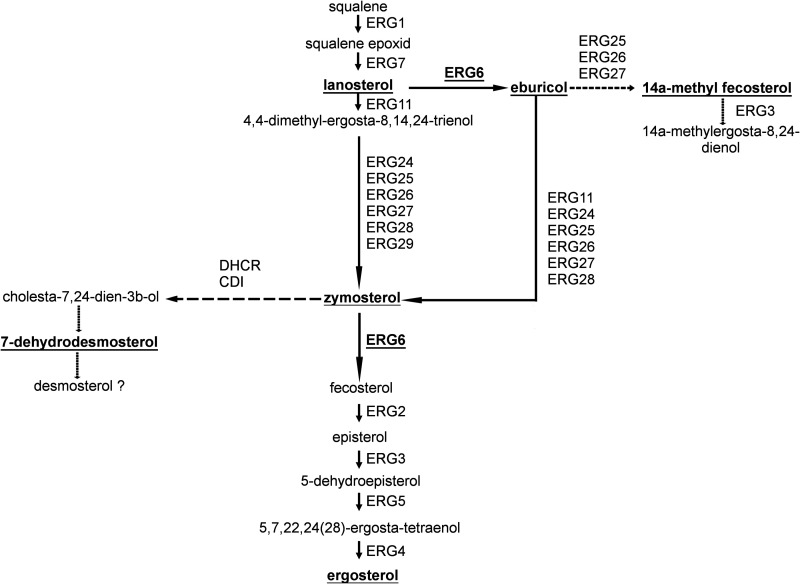
Possible ergosterol biosynthetic pathways of *M. lusitanicus*. Black arrows indicate the main ergosterol biosynthetic pathway, while dashed arrows indicate the alternative sterol biosynthetic pathways.

Previously, it was found in S. cerevisiae and Trichophyton rubrum that the formation of fecosterol (and thus finally of ergosterol) takes place via lanosterol and zymosterol (13, 45). In this case, the activity of Erg11 precedes the step catalyzed by Erg6. In A. fumigatus, C. neoformans, T. rubrum, and Fusarium keratoplasticum, however, analysis suggested that the Erg6-catalyzed formation of eburicol from lanosterol is part of the main ergosterol biosynthesis (37, 45–47). In this pathway, Erg6 converts lanosterol into eburicol, and this is only followed by the step catalyzed by Erg11. We assume that both pathways can be active in the *Mucor* cell at the same time, and if one of them is blocked, the flux of the ergosterol synthesis can shift to the other pathway (Fig. 11).

Figure 11 presents the proposed sterol biosynthesis pathways of *M. lusitanicus*. Analysis of the sterol composition in the wild type and the *erg6* knockout mutants indicated that ergosterol can be synthesized via two alternative pathways using lanosterol or eburicol as the precursor, respectively, and inhibition of *erg6b* can open up two further sterol pathways toward 7-dehydrodesmosterol and 14α-methylergosta-8-24(28)-dienol.

In S. cerevisiae, C. neoformans, and other fungi, an alternative pathway can be activated by the inhibition of Erg11 (13, 37). This can be achieved by azole treatment, since azoles inhibit lanosterol 14α-demethylase (Erg11) and cut the pathway toward zymosterol. In this case, lanosterol is converted by Erg6 to eburicol, and the alternative pathway produces the toxic 14α-methylergosta-8-24(28)-dienol. Production of this sterol analogue is thought to be one of the keys to the mechanism of action of azoles (13). In *M. lusitanicus*, inhibition of Erg11 led to the accumulation of a compound, of which the molecular mass and composition corresponded to those of 14α-methylfecosterol, the direct precursor of the toxic 14α-methylergosta-8-24(28)-dienol (Fig. 9). This result suggested the presence of this alternative route in *Mucor*. Presence of this pathway in mucormycosis-causing fungi was already raised, as accumulation of 14α-methylergosta-8-24(28)-dienol was reported in posaconazole-treated Rhizopus arrhizus (48). However, this compound could not be detected in our strains. This result may mean that the toxic endproduct of this biosynthetic side branch is not produced, or it can be present in very small amounts and/or only for a short time in *Mucor*, which may also contribute to the reduced effect of most azoles on these fungi.

Emergence of a new sterol product, 7-dehydrodesmosterol, in the *erg6b* knockout strain indicated that the blockage of *erg6* expression induced an alternative biosynthetic side branch starting from zymosterol (Fig. 9). In Mortierella alpina, belonging also to the *Mucoromycota* phylum of *Fungi*, 7-dehydrodesmosterol is converted to desmosterol, which was found to be the main sterol component in the membrane of this fungus (49, 50). However, our analysis did not detect desmosterol in any *M. lusitanicus* strains. This alternative pathway can also lead to the synthesis of cholesterol. However, cholesterol could be detected in all tested strains in a uniformly small amount. The facts that the genome of *M. lusitanicus* contains genes for enzymes that may be involved in further reactions of this pathway (i.e., DHCR and CDI) (51) and that knocking out the different *erg6* genes affected the transcription of these genes, suggested that this pathway may continue even further. Anyway, the fact that blockage of *erg11* and *erg6* leads to the formation of nontoxic azole compounds may contribute to the intrinsic azole resistance of *Mucor*.

Our experiments revealed that among the three *erg6* genes of *M. lusitanicus*, *erg6b* is the most relevant for sterol biosynthesis and it is crucial for the survival of *M. lusitanicus*. Moreover, expression of this gene seems to play a role in the pathogenicity of the fungus. A detailed analysis of the function of the *erg6* genes encoding 24-C-methyltransferase revealed four alternative sterol biosynthesis pathways in *M. lusitanicus*, which included two main parallel pathways leading to ergosterol. In these two pathways, the steps catalyzed by Erg11 and Erg6 enzymes follow each other in reverse order. The existence of several alternative pathways, their dynamic functioning, and the different sequences of the enzyme activities contribute fundamentally to the fact that mucormycosis-causing fungi can effectively avoid the effects of azoles. In addition, the presence of toxic sterols was not detected in response to azole treatments, which could also contribute to the limited effect of azoles.

## MATERIALS AND METHODS

### Strains, media, and growth conditions.

*Mucor lusitanicus* strain MS12 (*leuA*^−^ *pyrG*^−^) (52) and MS12+*pyrG* strains were used in the study. Fungal cultures were grown for 4 days under continuous light at 25°C. For nucleic acid extraction, we inoculated 10^6^ sporangiospores onto solid minimal medium [YNB; 10 g glucose, 0.5 g yeast nitrogen base without amino acids (Sigma-Aldrich), 1.5 g (NH_4_)_2_SO_4_, 1.5 g sodium glutamate, and 20 g agar per liter] supplemented with leucine and/or uracil (0.5 mg/mL), if required. RNA extraction was performed after cultivation in 10 mL RPMI 1640 without agar (Biosera), and inoculum size was 10^4^ sporangiospores/mL.

To measure the colony diameters of the strains, 10^4^ sporangiospores were inoculated onto the center of solid YNB plates for 4 days at 25°C. Colony diameters were measured daily using two technical and three biological replicates.

To determine the sporulation capacity of strains, 10^4^ spores were spread onto malt extract agar plates (MEA; 10 g glucose, 5 g yeast extract, 10 g malt extract, 20 g agar per liter). The plates were inoculated for 7 days at 25°C. After 7 days, all spores were washed from the surface of plates with phosphate-buffered saline (PBS; 137 mM NaCl, 2.7 mM KCl, 10 mM Na_2_HPO_4_, 2 mM KH_2_PO_4_ [pH 7.4]), and then the spores were centrifuged at 1,000 × *g* for 10 min and resuspended in 1 mL of PBS. The number of spores were counted with a Bürker chamber.

To determine the effects of membrane and cell wall stressors, 10^4^ spores were point inoculated at the center of YNB plates with or without stressor, which was SDS (4 mg/mL), Congo red (CR; 2 mg/mL), or calcofluor white (CFW; 0.1 mg/mL). For all growth tests, plates were cultivated at 28°C for 4 days in the dark, and colony diameter was measured daily. In each case, MS12-*pyrG* was used as the growth control.

### *In silico* analysis.

Homologous sequences of clinically relevant *Mucorales* species were searched on the JGI MycoCosm portal (https://mycocosm.jgi.doe.gov/mycocosm/home) (53). Nucleotide and amino acid sequences of Erg6 obtained from the *M. lusitanicus* CBS277.49v2.0 genome database and other clinically relevant *Mucorales* Erg6 sequences were aligned using the ClustalW program (54).

### Molecular techniques.

Genomic DNA and RNA samples were purified from mycelia using the ZR fungal/bacterial DNA MiniPrep system (Zymo Research) and the Quick-RNA MiniPrep kit (Zymo Research), respectively, following the manufacturer's instructions.

Genes were amplified by PCR using the Phusion Flash high-fidelity PCR master mix (Thermo Scientific) and the primers presented in Table S1. Primer and oligonucleotide sequences were designed using the *M. lusitanicus* CBS277.49v2.0 genome database.

### Real-time RT-qPCR analysis.

Reverse transcription was carried out with the Maxima H Minus first-strand cDNA synthesis kit (Thermo Scientific) using random hexamer and oligo(dT)_18_ primers, following the instructions of the manufacturer. The qRT-PCR experiments were performed in a CFX96 real-time PCR detection system (Bio-Rad) using the Maxima SYBR green qPCR master mix (Thermo Scientific) and the primers presented in Table S1. The relative transcript levels of genes were determined with the threshold cycle (2^−-ΔΔ^*^CT^*) method (55) using the actin gene of *M. lusitanicus* as a reference (33). Experiments were performed in biological and technical triplicates.

### Design of gRNAs and construction of deletion cassettes to create *erg* knockout mutant strains.

The protospacer sequences designed to target the DNA cleavage site in *erg6a* (CBS277.49v2.0 genome database protein ID 74496), *erg6b* (CBS277.49v2.0 genome database protein ID 155859), and *erg6c* (CBS277.49v2.0 genome database protein ID 151310) genes were the following: 5′-TGCCATGCTCCCTCCTTTGA-3′, 5′-CACTATTTAGCCGCCAACAT-3′, and 5′-AGATTACACTAGCTTCATCA-3′, respectively. Using these sequences, the Alt-R CRISPR crRNA and Alt-R CRISPR-Cas9 tracrRNA molecules were designed and purchased from Integrated DNA Technologies (IDT). To form the crRNA:tracrRNA duplexes (i.e., the guide RNAs [gRNAs]), nuclease-free duplex buffer (IDT) was used according to the instructions of the manufacturer. Genome editing strategies followed the setup described earlier (56). Homology-directed repair (HDR) was applied for all gene disruptions following a strategy described previously (56). Disruption cassettes functioning also as the template DNA for the HDR were constructed by PCR using the Phusion Flash high-fidelity PCR master mix (Thermo Scientific). At first, two fragments, upstream from the start codons and downstream from the stop codons of the targeted gene and the *M. lusitanicus pyrG* gene (CBS277.49v2.0 genome database protein ID 36136) or *M. lusitanicus leuA* gene (CBS277.49v2.0 genome database protein ID 33992) along with its own promoter and terminator sequences were amplified using gene-specific primer pairs (see Table S1 in supplemental material). The amplified fragments were fused in a subsequent PCR using nested primers (Table S1); the ratio of the fragments in the reaction was 1:1:1.

### Transformation experiments.

For CRISPR-Cas9-mediated gene knockout, polyethylene glycol-mediated protoplast transformation was used according to the method of van Heeswijck and Roncero (57). Protoplasts were prepared as described earlier (58). For the gene disruption by the CRISPR-Cas9 method, 5 μg template DNA (i.e., the disruption cassette), 10 μM gRNA, and 10 μM Cas9 nuclease were added to the protoplasts in one transformation reaction mixture, as described by Nagy et al. (56). In each case, transformants were selected on solid YNB medium by the complementation of the uracil and/or leucine auxotrophy of the MS12 strain. From each primary transformant, monosporangial colonies were formed under selective conditions. In case of the successful transformation experiments, we used PCR analysis of the mutant strains amplifying the expected fragments using nested primers (McErg6aP7-McErg6aP8, McErg6bP7-McErg6bP8, and McErg6cP7-McErg6cP8 [Table S1]) (Fig. S1C). Mutants proved to be mitotically stable, retaining the integrated fragment even after 20 cultivation cycles. qRT-PCR analysis proved the absence of the transcripts of the disrupted genes. For further analysis, two independently derived mutants were selected.

### Susceptibility tests.

Sensitivity of the fungal strains to different antifungal agents was examined in a 96-well microtiter plate assay. The susceptibility test was performed according to the CLSI recommendation (59) in three biological replicates. Ketoconazole (Alfa Aesar), itraconazole (Across Organics), fluconazole (Alfa Aesar), ravuconazole (Sigma-Aldrich), posaconazole (Sigma-Aldrich), isavuconazole (Sigma-Aldrich), and amphotericin B (Sigma-Aldrich) were dissolved in dimethyl sulfoxide to prepare the stock. These stocks were then diluted with liquid RPMI 1640 medium. Final concentrations of azoles and AmB in the wells ranged from 0.125 to 16 μg/mL. Inocula were prepared and diluted in liquid RPMI 1640. Plates were incubated for 48 h at 25°C. For the antifungal susceptibility test, Candida krusei ATCC 6258 was used as a reference strain.

### Survival assay in Galleria mellonella larvae.

For the G. mellonella larvae assays, spores were resuspended in insect physiological saline (IPS); (50 mM NaCl, 5 mM KCl, 10 mM EDTA, and 30 mM sodium citrate in 0.1 M Tris-HCl [pH 6.9]) (60). G. mellonella larvae (TruLarv; BioSystems Technology) were inoculated with 10^5^ fungal spores in 20 μL IPS via the last proleg using 29-gauge insulin needles (BD Micro-Fine). For each *M. lusitanicus* strain, 20 larvae were infected. For IPS-treated (uninfected) and witness controls (no injections, uninfected), 20 animals were utilized. Larvae were maintained at 30°C, and their survival was monitored daily for 6 days. The results shown are representative of at least three independent experiments using two independent isolates per mutant.

### Analysis of sterol composition using HPLC-HRMS.

Approximately 10 mg of freeze-dried mycelia was sonicated with 2 mL of 10% (mass/vol) KOH in methanol for 5 min. The sterols were deliberated by saponification at 80°C for 90 min. After cooling down to room temperature, 500 μL of water, 1 mL of *n*-hexane, and 5 μL of β-sitosterol (1 mg/mL; internal standard) were added to the mixtures, followed by vigorous vortexing for 30 s. The upper hexan layer was transferred to a 4-mL amber vial. The extraction with hexane was repeated twice, and the combined organic phases were evaporated to dryness under a gentle stream of nitrogen. The residues were dissolved in 500 μL of methanol and filtered through a 0.22-μm polytetrafluoroethylene membrane filter. Five microliters of the final solution was injected into the HPLC-HRMS system.

LC-HRMS measurements were performed using a DionexUltimate 3000 UHPLC system coupled to an Q Exactive Plus hybrid quadrupole-Orbitrap mass spectrometer operating with an atmospheric pressure chemical ionization source. Sterols were separated using a Gemini-NX-C_18_ column (3 μm, 150 by 2 mm) with thermostat at 40°C. For gradient separation, water (A) and methanol (B) served as mobile phases at a flow rate of 0.4 mL/min. The gradient elution was as follows: 0.0 min, 95% B; 1.0 min, 95% B; 4.0 min, 100% B; 7.0 min, 100% B; 7.5 min, 95% B; 10.0 min, 95% B.

All samples were analyzed in positive ionization mode. The ion source had the following settings: the temperature of the probe heater and ion transfer capillary, spray current, sheath gas flow rate, auxiliary gas flow rate, and S-lens RF level were set to 350°C, 300°C, 4.0 μA, 30 arbitrary units, 5 arbitrary units, and 50 arbitrary units, respectively.

The mass spectrometer acquired data using a full-scan, data-dependent tandem MS (MS/MS) method (full MS/ddMS2). The full-scan MS spectra were acquired at a resolution of 70,000 from *m/z* 200 to 550 with a maximum injection time of 100 ms. For every full scan, 5 ddMS2 scans were carried out with a resolution of 17,500 and a minimum automatic gain control target of 1.00 × 10^6^. The isolation window was 0.4 *m/z*. Under the applied condition, dehydrated molecular ions [M+H-H2O]^+^ of each of the sterols were observed. HPLC-HRMS data were acquired using Trace Finder 4.0 software. The raw MS data files were processed using Thermo Xcalibur (4.0) software. To identify the sterols, the following standards were used: cholesterol, ergosterol, lanosterol, euphorbol (which is a stereoisomer of eburicol), and zymosterol from Sigma-Aldrich.

Regarding the structure, the only difference between desmosterol and 7-dehydrodesmosterol is the degree of unsaturation within the B-ring, which leads to the formation of different ions by the fragmentation within the C-ring and the loss of the side chain. As reported Münger et al. (61), higher intensities of *m/z* 145 and *m/z* 159 are characteristic for Δ5,Δ7 sterols (7-dehydrodesmosterol), while for Δ5 sterols (desmosterol), the predominant species are *m/z* 147 and *m/z* 161. In addition, the loss of the side chain leads to *m/z* 257 for Δ5 sterols (desmosterol), whereas *m/z* 255 can be observed for Δ5,Δ7 sterols (7-dehydrodesmosterol) (Fig. S4).

### Statistical analysis.

All measurements were performed in two technical and three biological replicates. Significance was calculated with paired *t* test or one-way analysis of variance (ANOVA) using the GraphPad Prism 7.00 program (GraphPad Software, La Jolla, CA, USA). *P* values of <0.05 were considered statistically significant.
