# Supplementary material for: Characterization of the Sterol 24-C-Methyltransferase Genes Reveals a Network of Alternative Sterol Biosynthetic Pathways in Mucor lusitanicus
Source: Microbiol Spectr. 2023 Apr 10;11(3):e00315-23. doi: 10.1128/spectrum.00315-23 (PMC10269636; doi:10.1128/spectrum.00315-23)
Supplement: Supplemental file 1 — Fig. S1 to S4 and Table S1. Download spectrum.00315-23-s0001.pdf, PDF file, 1.0 MB [file spectrum.00315-23-s0001.pdf]

Supplemental materials

Characterization of the sterol 24-C-methyltransferase genes reveals a network of alternative sterol biosynthetic pathways in *Mucor lusitanicus*

Kitti Bauer[a,b], Bence Rafael[a,b], Bernadett Vágó[a,b], Sándor Kiss-Vetráb[a,b], Anna Molnár[a,b], Csilla Szebenyi[a,b], Mónika Varga[a], András Szekeres[a], Csaba Vágvölgyi[a,b], Tamás Papp[a,b*¶], Gábor Nagy[a,b*¶]

[1]Department of Microbiology, University of Szeged, Szeged, Hungary

[2]ELKH-SZTE Fungal Pathomechanisms Research Group, Faculty of Science and Informatics, University of Szeged, Szeged, Hungary

*Corresponding authors

E-mail: pappt@bio.u-szeged.hu (T.P.); or nagy.gabor.04@szte.hu (G.N.)

¶ These authors contributed equally to this work

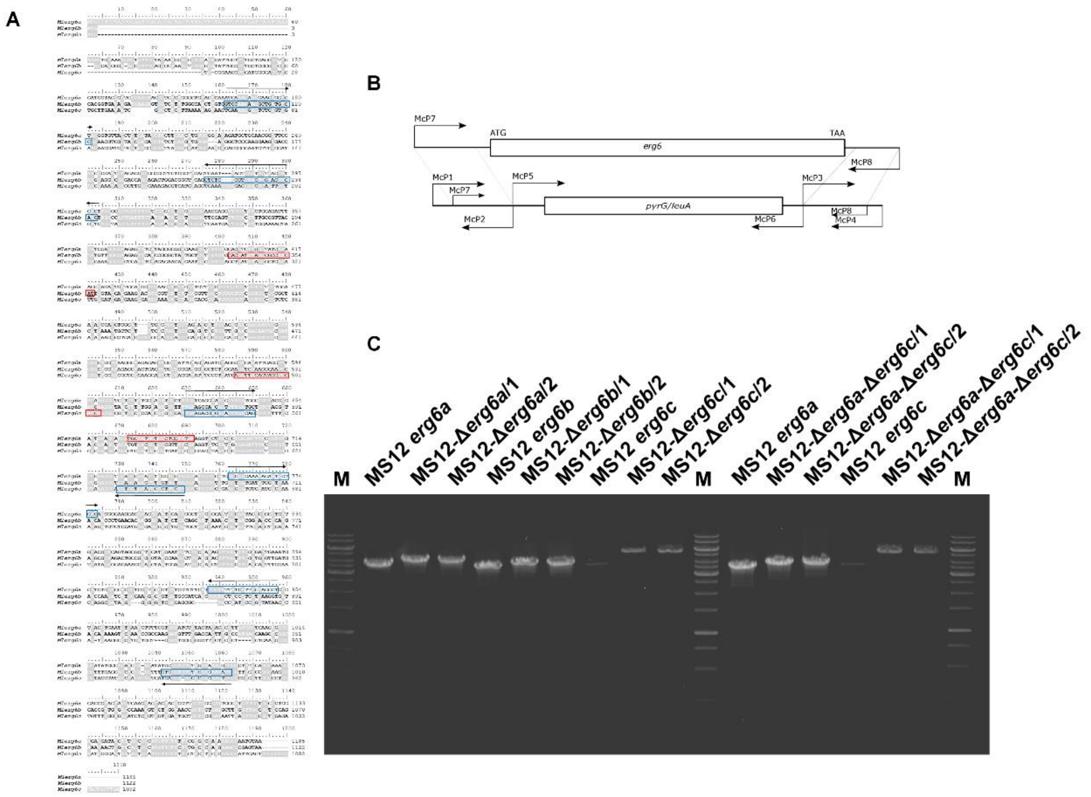

FIG S1 (A) Nucleotid alignment of *M. lusitanicus* erg6b genes. Red boxes indicate the protospacer sequences, blue boxes indicate the specific real-time primers binding sites, while the arrows shows the orientation of primers (B) Genome editing strategy designed to disrupt the *erg6* genes of Mucor lusitanicus using the CRISPR-Cas9 method. HDR was performed using the disruption cassette/template DNA containing either the *pyrG* or the *leuA* gene as selection markers. Positions of the primers used to analyze or amplify the constructs are presented (for the nucleic acid sequences of the primers, see Supplementary Table S1). (C) PCR analysis of the transformants. For gele pictures M: GeneRuler 1 kb DNA ruler (Thermo Scientific), MS12 was the parental strain.

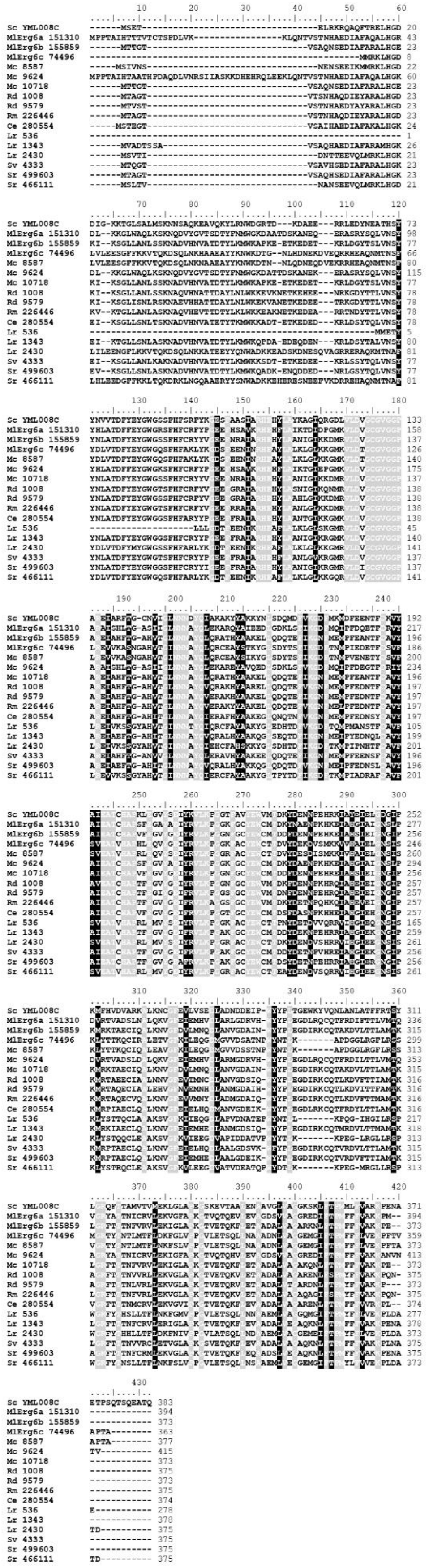

	Ml Erg6a	Ml Erg6b	Ml Erg6c	Mc 8587	Mc 9624	Mc 10718	Rd 1008	Rd 9579	Rm 226446	Ce 280554	Lr 536	Lr 1343	Lr 2430	Sv 4333	Sr 499603	Sr 466111
Ml Erg6a	–	62.87 %	41.53 %	41.11 %	88.26 %	62.33 %	61.52 %	61.62 %	62.06 %	65.32 %	43.07 %	61.44 %	41.02 %	65.05 %	63.14 %	38.73 %
Ml Erg6b		–	47.65 %	47.31 %	63.59 %	99.20 %	81.82 %	81.23 %	80.48 %	81.13 %	48.91 %	81.45 %	46.22 %	83.33 %	80.43 %	45.74 %
Ml Erg6c			–	93.94 %	41.64 %	47.37 %	45.60 %	46.15 %	45.60 %	45.75 %	72.69 %	45.05 %	72.7%	44.66 %	45.05 %	73.26 %

FIG S2 Amino acid alignment of Erg6 proteins of clinically relevant Mucorales species. Grey background shows the same amino acids, while black background shows the similar amino acids. Number indicates the protein ID of Erg6 proteins. The table indicate the percentage of similarity between *Mucor lusitanicus* Erg6 proteins and other Mucorales Erg6 proteins. Sc: *Saccharomyces cerevisiae*; Mc-*Mucor circinelloides*; Rd-*Rhizopus delemear*; Ce-*Cunninghamella echinulata*; Lr-*Lichtheimia ramosa*; Sv-*Saksanaea vasiformis*; Sr-*Syncephalastrum racemosum*

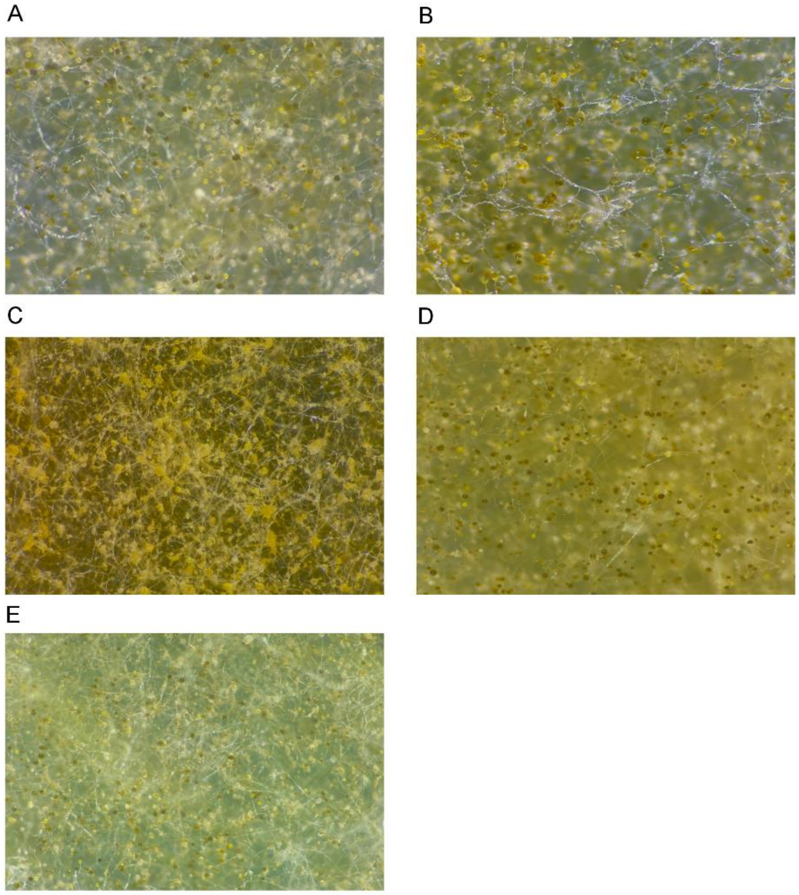

FIG S3 Sporangia of of *erg6* mutants and MS12+*pyrG* strain under stereo microscope (A) MS12+*pyrG* (B) MS12-Δ*erg6a* (C) MS12-Δ*erg6b* (D) MS12-Δ*erg6c* (E) MS12-Δ*erg6a*-Δ*erg6c*.

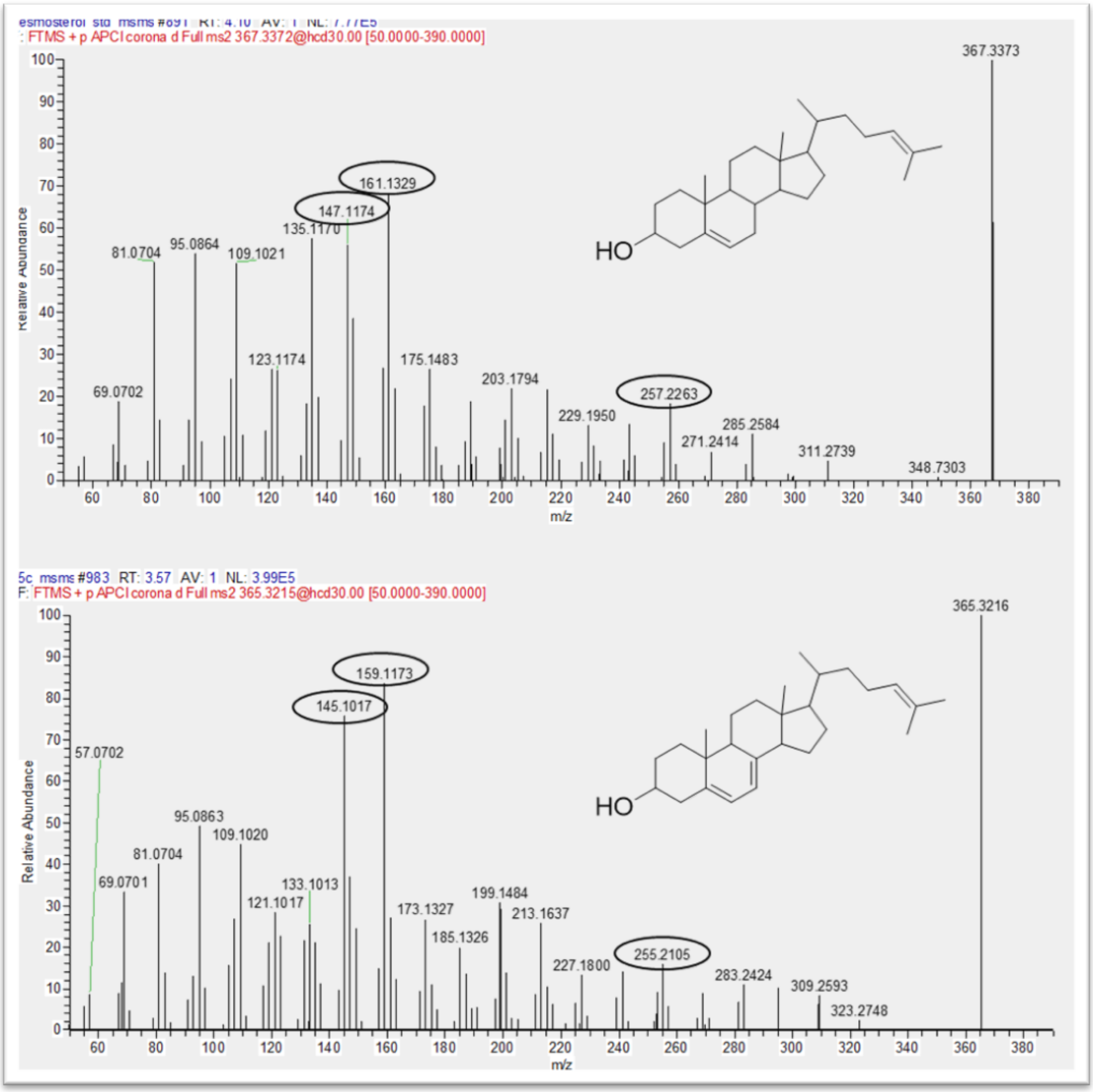

FIG S4 MSMS spectra of desmosterol standard and the putative 7-dehydrodesmosterol.

TABLE S1 Primers used in the present study.

Primers	Sequence 5’-3’	Amplified DNA
	**Phusion PCR for *erg6a* deletion**	
McErgabP1	GTTCAAGCCGCCTTGTTCTG	Promoter of *erg6a*
McErg6aP2	TAGTCTGCCTCCGTATCCGA	
McErg6aP3	TGAACTAGGCCTCAAATACC	Terminator of *erg6a*
McErg6aP4	AAATCGCTACTTTGGGTCTG	
McErg6apyrGP5	CTCCGCCTGAAAATCGGATACGGA GGCAGACTATGCCTCAGCATTGGTACTTG	*pyrG* with own promoter and terminator
McErg6apyrGP6	CTCGTAATCTAACGGTATTTGAGGC CTAGTTCAGTACACTGGCCATGCTATCG	
McErg6aleuAP5	CGCCTGAAAATCGGATACGGAGGCA GACTACTGCAGGATGGGACAAGGTATATAT	*leuA* with own promoter and terminator
McErg6aleuAP6	GTAATCTAACGGTATTTGAGGCCTA GTTCACTGCAGTAGCTGTTGATGTTGTTGT	
McErg6aP7	TTGCGTATGGATTCTTGTTTCCTC	Final PCR product for transformation
McErg6aP8	TTAATCACACTTCGATTCCCGT	
	**Phusion PCR for *erg6b* deletion**	
Mc155859P1	CTGGAATCTGTCAACTTAACCC	Promoter of *erg6b*
Mc155859P2	CATCTGTCTCAATATCCGTCGT	
Mc155859P3	CTTCAAGGGGGGACGACGGATATTG AGACAGATGTGCCTCAGCATTGGTACTTG	*pyrG* with own promoter and terminator
Mc155859P4	GGAACAGGAGAGATTTGATGGTGG TGTTGTAAGGGTACACTGGCCATGCTATCG	
Mc155859P5	CCTTACAACACCACCATCAAATCTC	Terminator of *erg6b*
Mc155859P6	TTTGCCCGCTGTAGATGATACC	
Mc155859P7	TGTGGCGCTCAATTCTACTG	Final PCR product for transformation
Mc155859P8	ACATTTGCACCCTTCTTTCC	
	**Phusion PCR for *erg6c* deletion**	
Mc151310P1	GAATGCTATCTGCTGAATCTG	Promoter of *erg6c*

Mc151310P2	TTTGTTTCTGGACTGATGCT	
Mc151310P3	TTGTCGATCATCTCAACTCAG	Terminator of
Mc151310P4	CGTTAAATGCCGCTAGATCAC	*erg6c*
Mc151310P5	GATATTTAAAAGCATCAGTCCAGAA ACAAACTGCAGGATGGGACAAGGTATATAT	*pyrG* with own promoter and terminator
Mc151310P6	ATAACGCCGCTGAGTTGAGATGATC GACAACTGCAGTAGCTGTTGATGTTGTTGT	
Mc151310P7	CCAGCATATCCGATGATCTC	Final PCR product
Mc151310P8	TGCTAGATGAACAACTACAGAC	for transformation
	**Primers for qRT-PCR experiments**	
Mc74496rtfw	ACCGACAAATACAATGCTGAG	*erg6a*
Mc74496rtrev	ACCCTCCAAAGGATAATACCA	
Mc155859rtfw	GTCCAAAGAACGCTGATGTCC	*erg6b*
Mc155859rtrev	GTTGTAGTACGAGTTGACGAGAGAG	
Mc151310rtfw	TGAAGACGAGACATTTGACAG	*erg6c*
Mc151310rtrev	TAGCAAGCGAATTTACCACC	
Mc157152rtfw	GATGCTCAACACTACAAGATCC	*erg2*
Mc157152rtrev	CAATTCACCAACGATAGCCT	
Erg3afw	GCTCACTCTTCCCTTCTTCAC	*erg3a*
Erg3arev	CTGATTGTGGTATCTACTGGTTCC	
McP450/2_fw	GGGTAATTTCCTCTGTGCTG	*cyp51a*
McP450/2_rev	CTACTTCCAGTTGATGAACGG	
McP450/1_fw	TTCGGTGCTGTTGGTATCTC	*cyp51b*
McP450/1_rev	GGAAGTGTAATCAGGCTTAGGA	
Erg4afw	GCAACAAGACAAACAAGAATGG	*erg4a*
Erg4arev	AGGACGAGTTTGGGTGTTAC	
Erg4bfw	GCAACAAGACAAACAAGAATGG	*erg4b*
Erg4brev	AGGACGAGTTTGGGTGTTAC	
Erg5afw	GCTTTAGGTGGTGTTTCAGAG	*erg5a*
Erg5arev	CCATCCAATCTCGCAAATACA	

Erg5bfw	ATTCATTGTCATCGCATCCA	*erg5b*
Erg5brev	GGCAAGGAACACAACAACTA	
Erg25afw	CTCGCCACTTTATCCCTTTCTG	*erg25a*
Erg25arev	GAAGGTAATCCCACCAACGG	
Erg25brev	GTGTTCAAATTAGACGATGCTTGG	*erg25b*
Erg25brev	AGAAGGGAACCCAGTTACAGAG	
McErg26rtFw	CAAGCCTTCAATTTAACCAACGG	*erg26*
McErg26rtRev	TCACTAATAAGAGCAATCACCAGAC	
McErg27rtFw	ATTAGAAGCAGAAGGAGAAGGT	*erg27*
McErg27rtRev	GCAGATGAGAACGACAAGAC	
Erg28rtfw	ACTTATGTGATTGCCTTGACC	*erg28*
Erg28rtrev	TGACTTGAGTGAGCCAAATGAG	
MlDHCR7artfw	CTGTCATCCAAACCAGTCCT	*dhcr7a*
MlDHCR7artrev	CCTTCATATCCGAGCCACTG	
MlDHCR7brtfw	AAGAGTAGCAGTTGGTTGGG	*dhcr7b*
MlDHCR7brtrev	TCTCAATGATCGAGGGTCCA	
MlDHCR7crtfw	CTTCAACCTACCAGCAATACAG	*dhcr7c*
MlDHCR7crtrev	ACTATCAATGCACAATCAGGG	
MlDHCR7drtfw	CCGAGATGCTGTTTCAACCC	*dhcr7d*
MlDHCR7drtrev	CTGTCCTCCTTGTCTTGATCCA	
MlCDIrtfw	ATGACAGCACTTGTTGGTATGAG	*cdi*
MlCDIrtrev	GGCGTATTCCTTCCATAGTTGAG	
McactinF	CACTCCTTCACTACCACCGCTGA	actin
McactinR	GAGAGCAGAGGATTGAGCAGCAG	
